# Effect of Rice Husk and Wood Flour on the Structural, Mechanical, and Fire-Retardant Characteristics of Recycled High-Density Polyethylene

**DOI:** 10.3390/polym15194031

**Published:** 2023-10-09

**Authors:** Atta Ur Rehman Shah, Abdul Jalil, Atiya Sadiq, Meshal Alzaid, Muhammad Shoaib Naseem, Rakan Alanazi, Sultan Alanazi, Abdullatyf Obaid Alanzy, Ibrahim Hotan Alsohaimi, Rizwan Ahmed Malik

**Affiliations:** 1 Department of Mechanical Engineering, COMSATS University Islamabad, Wah Campus, Wah Cantt 47040, Pakistan; 2 Department of Mechanical Engineering, HITEC University, Taxila 47050, Pakistan; 3 Department of Physics, College of Science, Jouf University, Sakaka P.O. Box 2014, Al-Jouf, Saudi Arabiaalzyrbdalltyf@gmail.com (A.O.A.); 4 Department of Chemistry, College of Science, Jouf University, Sakaka P.O. Box 2014, Al-Jouf, Saudi Arabia; ehalshaimi@ju.edu.sa; 5 Department of Metallurgy & Materials Engineering, Faculty of Mechanical and Aeronautical Engineering, University of Engineering and Technology, Taxila 47050, Pakistan; rizwan.malik@uettaxila.edu.pk

**Keywords:** rice husk, polymer composites, wood flour, recycled high-density polyethylene

## Abstract

Given the rising consumption of plastic products, it is becoming imperative to prioritize the recycling of plastic items as a solution to reducing plastic waste and environmental pollution. In this context, this research focuses on assessing the impact of incorporating rice husk and wood flour into recycled high-density polyethylene (rec-HDPE) to analyze its mechanical properties, flammability, and thermal stability. The combined rec-HDPE content of wood flour and rice husk varied between 0% and 20%. The rec-HDPE content of maleic anhydride grafted polyethylene (MAPE) was fixed at 3%. Mechanical characteristics such as flexural, tensile, and impact strengths were assessed. Cone calorimetry (CC) tests, limited oxygen index (LOI) tests, and horizontal and vertical burning tests were performed to determine the flammability or fire retardancy of these composites. On the other hand, to characterize the thermal characteristics of these composites, thermogravimetric analysis (TGA) was used. To further characterize the fluctuation in these characteristics, scanning electron microscopy (SEM) and infrared spectroscopy (FTIR) studies were carried out. The mechanical characteristics were found to be increased in response to adding rice husk or wood flour. An 8% increase in tensile strength and a 20% increase in elastic modulus enhancement were recorded for a 20% rice husk-added composite. SEM revealed the reason for the variation in tensile properties, based on the extent of agglomeration and the extent of uniform distribution of fillers in rec-HDPE. Following these lines, the 20% rice husk-added composite also showed a maximum increase of around 6% in its flexural strength and a maximum increase of 50% in its flexural modulus. A decrease in impact strength was recorded for rice husk and wood flour-reinforced composites, compared with unreinforced rec-HDPE. Hybrid composites displayed a lack of mechanical strength due to changes in their nature. FTIR tests were performed for a much more elaborate analysis to confirm these results. Twenty percent of rice husk-added rec-HDPE displayed the best thermal properties that were tested, based on TGA and derivative thermogravimetric (DTG) analysis. This 20% composite also displayed the best fire-retardancy characteristics according to UL 94 tests, cone calorimetry tests, and limited oxygen index tests, due to the barrier created by the silica protective layer. These tests demonstrated that the incorporation of both fillers—rice husk and wood flour—effectively enhanced the thermal, mechanical, and fire-retardant attributes of recycled HDPE.

## 1. Introduction

Despite the extensive research and development devoted to composite materials, due to their remarkable properties and versatility across various applications [1,2,3], there exists a noticeable gap in the study of recycled materials for the development of new composites. Recycling materials have assumed paramount significance in our modern world, as they provide a crucial avenue for promoting environmental sustainability and responsible resource management [4,5]. As the consumption of plastic products is rapidly increasing, there is a great need to recycle plastic products and use such recycled products to reduce plastic waste and environmental pollution [6]. For this purpose, fillers, especially bio-fillers, are the best option for converting plastic waste products into useful composite materials. Composite materials are combinations of two or more materials that may be chemically and physically different from each other but, when combined, can result in a product that has desirable properties and that can be used for specific tasks.

The hybridization of polycarbonate (PC) in wood flour/high-density polyethylene composites resulted in improved mechanical properties and fire-retardancy characteristics [7]. The mechanical properties were studied in terms of tensile modulus, tensile strength, flexural strength, and flexural modulus. Flame retardancy was observed by means of cone calorimetry. On the other hand, thermal stability was slightly improved by inserting rice husk filler into a recycled high-density polyethylene/polyethylene terephthalate blend, with an increase in the derivative thermogravimetric (DTG) degradation temperature [8].

Arjmandi et al. [9] prepared rice husk-filled biocomposites. They added rice husk as a filler in different polymer matrices including polypropylene, polyethylene, polylactic acid, and polyvinyl chloride to develop polymer composites and studied the effects on the composites’ mechanical properties. Rice husk was added to polypropylene to reinforce the polymer matrix and its thermal stability was investigated. The results showed that the heat-deflection temperature and the degradation temperature of the rice husk polypropylene composite were improved, thus increasing its thermal stability [10]. Moreover, adding rice husk particles to the polymer matrix enhanced the melting-temperature range, which showed that rice husk, in the mixture, has some degree of thermal resistance to melting [11]. Zhao et al. investigated the flame retardancy of rice husk-filled high-density polyethylene (HDPE) composites [12]. They added varied amounts of rice husk into the HDPE matrix. The thermo-oxidation process was successfully delayed by 40 °C with the addition of rice husk. A blend of rice husk and polypropylene was prepared and investigated. The result confirmed that with the addition of rice husk particles, the thermal stability of the polymer matrix was enhanced [13]. It was also discovered that rice husk particles enhanced the fire-retarding properties of composites, in addition to reducing dripping, due to the protective role played by the husk particles on the polymer matrix [10]. The thermal properties of HDPE/rice husk composites were also enhanced by increasing maleic anhydride grafted polyethylene (MAPE) content [14]. Pandey et al. developed a hybrid composite made with softwood biochar, sisal fiber, and epoxy as resin [15]. The maximum flexural, tensile, and impact strengths were found with 10%, 5%, and 15% of biochar content, respectively. Similarly, Ketabchi et al. carried out an analysis of variant biochar-reinforced polypropylene/ethylene-vinyl acetate hybrid composites [16]. They found that a 30% biochar-reinforced composite showed enhanced mechanical characteristics, without affecting thermal properties.

Mechanical properties are dependent on a large number of factors. The size of filler particles plays an important role in determining the mechanical properties of composite materials [17]. Coarse and larger filler particles usually result in low strength due to less surface area and larger stress concentration. The smaller the size of filler particles, the more strength they will provide to the composite material because, at a smaller size, a greater number of particles share the applied stress [18]. Dispersion of nanoparticles also has a huge impact on the mechanical characteristics of composite materials and plays a very prominent role in varying their properties [19]. When particles are evenly dispersed, they will impart greater strength to the composite material. Interfacial bonding between atoms of the polymer matrix and nanoparticles also has an impact on the mechanical characteristics of the composite material, which cannot be neglected [20]. Moreover, better interfacial bonding between nanoparticles and the matrix results in greater strength and much more improved mechanical characteristics of the composite material. On the other hand, a coupling agent can be used for better interfacial bonding. A coupling agent is added in very small amounts. It provides more adhesion between nanoparticles and the polymer matrix, which leads to improved mechanical properties [21].

It makes the filler react with the atoms of the polymer, due to which bonding between the filler and polymer atoms strengthens. As a result, its mechanical properties increase. When wood flour was mixed with acrylic acid as a coupling agent, yield strength and elongation at break decreased, while tensile modulus and flexural strength increased. When wood flour was mixed with HDPE along with maleic acid as a coupling agent, all the properties, including yield strength, elongation at break, tensile modulus, and flexural strength, increased [22]. On the other hand, rice husk was mixed with HDPE to prepare biomaterials to promote the utilization of agricultural waste [23,24]. Bending strength was found to be high in HDPE by a using silane coupling agent. This shows that a coupling agent could greatly affect the mechanical properties. It modifies the interfacial properties of the composite and increases the interfacial adhesion of fillers and the matrix, which in turn enhances mechanical properties [25]. Interfacial bonding also played a vital role in the mechanical properties of wood–plastic composites [26]. Interfacial bonding of the composite is enhanced by a compatibilizer, thereby improving the mechanical and creep resistance of the composite [27,28].

While there has been substantial research conducted on composite materials using HDPE, there is a dearth of studies specifically addressing recycled high-density polyethylene (rec-HDPE)-based composites. In this scenario, this study aims to create and explore a novel composite material by combining recycled high-density polyethylene, a versatile and durable plastic, with rice husk and wood flour, two abundant agricultural byproducts. This paper investigates the potential of these components to enhance the composite’s properties and lead to innovative and environmentally sustainable materials suitable for various applications. The prime aim of this research is to enhance the mechanical, fire retarding, and thermal properties of recycled high-density polyethylene by incorporating rice husk and wood floor into it.

## 2. Materials and Methodology

### 2.1. Materials

Recycled HDPE pellets, having a density of 0.96 g/cm^3^ and a melt flow index of 0.55 g/10 min, were obtained from NUTECH pipes in Sangjani, Rawalpindi, Pakistan. Rice husk with a bulk density of 0.12 g/cm^3^ was purchased from Allied Industry in Lahore, Pakistan. Wood flour with a bulk density of 0.21 g/cm^3^ was purchased from the National Plywood Center in Rawalpindi, Pakistan. Maleic anhydride polyethylene (MAPE) with a melt flow index of 1.53 g/10 min was obtained from Sitara Chemicals in Faisalabad, Pakistan.

### 2.2. Fabrication of Composites

A masala grinder, specifically a Geepas masala grinder with an RPM of 4500, was used to make rice husk powder. The obtained rice husk powder was then sieved with a mesh size of 50 to reduce the particle size of the rice husk powder to less than 300 µm. Wood flour was received in an already crushed form, and to reduce the size to below 500 µm, it was sieved with a mesh size of 35. Rice husk, wood flour, and recycled HDPE were dried for 36 h under sunlight to eliminate any possible moisture. Recycled HDPE was then mixed with rice husk and wood flour for 30 minutes in a high-speed mixer to achieve uniform blends as specified in Table 1. A micro twin screw extruder (SHJ-Omega-20) with a screw outer diameter of 19.8 mm and a length/diameter ratio of 38:1 was utilized to mix these homogeneous composite blends. The extruder’s extruding and mixing zones were both set to 180 °C. These pellets were then placed in a manual injection molding machine to create a variety of samples for mechanical and other tests. The manual injection molding temperature was set at 185 °C.

### 2.3. Characterizations

**Figure 1:**
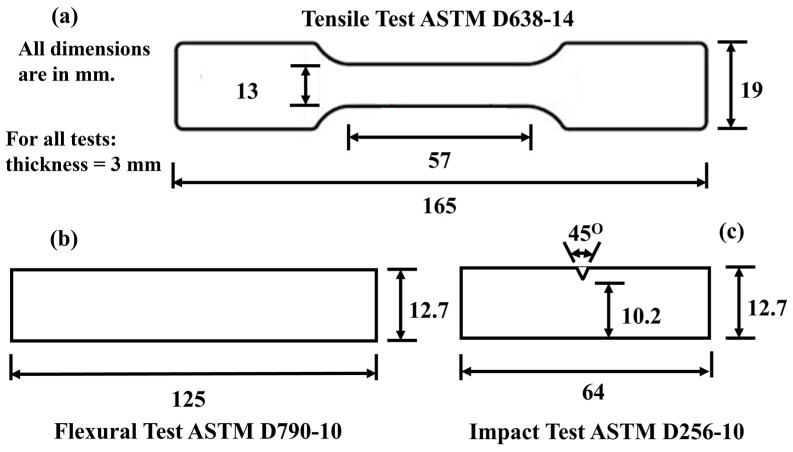
Schematic of (**a**) tensile test specimen, (**b**) flexural test specimen, (**c**) impact test specimen.

The possible interactions between the matrix (rec-HDPE), rice husk, and wood flour were investigated and analyzed using FTIR. This was accomplished using FTIR equipment (Scientific Nicolet 6700 model) with a KBr disc method, and the spectra spanning from 400 to 4000 cm^−1^. The powder samples (rec-HDPE, rice husk, and wood flour) were mixed with an appropriate amount of KBr followed by compression to make pellets. Impact, tensile, and flexural tests were all used to characterize the samples mechanically. Tensile testing was carried out using Testometric Inc., Manchester, UK (load cell: 100 kN), with a cross-head speed of 1.5 mm/s following ASTM standard D638-14, as shown in Figure 1a [24]. The three-point flexural test was accomplished using the Testometric Inc., UK with a cross-head speed of 1.5 mm/s, following ASTM standard D790-10, as shown in Figure 1b [24]. TM235 (load arm: 16 kg Model) was utilized to perform the Izod impact test following ASTM standard D256-10, as shown in Figure 1c [24].

SEM was performed on specimens that underwent tensile testing using a JOEL JSM5910 microscope from Tokyo, Japan. This microscope was used to analyze the basic causes and reasons behind the variations in tensile properties. Horizontal and vertical burning (UL-94), cone calorimetric (CC) tests, and the limited oxygen index (LOI) tests were all used to assess the fire resistance of all composites. ASTM standard D635-03 was used to perform the horizontal burning test that exhibited the findings of burning time and rate of different composites [24]. Based on that, an HB rating was given to them. On the other hand, ASTM standard D3801-19 was employed to perform the vertical burning test [24]. In the vertical burning test, measurements were taken for cotton dripping due to ignited particles, after-flame time, afterglow time, and whether the flame had reached the holding clamp or not. Based on the above-mentioned parameters, composites were awarded ratings of V-0, V-1, V-2, or no rating. The CC test was performed in accordance with ASTM standard 1354-17 in FTT Limited, East Grinstead, with an external heat flux of 50 kW/m^2^ [24]. The LOI test was performed in a station-limited oxygen index tester as per ASTM standard D2863-17. Oxygen and oxygen–nitrogen mixture flow rates were 3 L/min and 20 L/min, respectively [24].

Thermal stability was analyzed using TG curves on the Perkin Elmer (Pyris Diamond Series TG/DTA model). The sample was heated at a constant rate in an inert atmosphere of nitrogen with a flow rate of 35 mL/min. The heating was carried out at a rate of 20 °C/min.

## 3. Results and Discussion

### 3.1. FTIR

**Figure 2:**
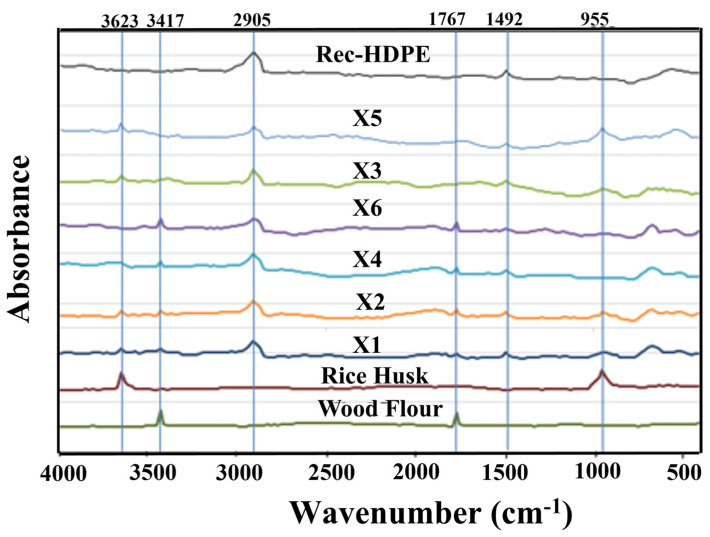
FTIR spectrum of all samples.

The FTIR spectra of all prepared composites are shown in Figure 2. Various peaks are detected at different wavenumbers. These peaks vary in intensity from sample to sample. The C-H stretching vibration which is ascribed by aliphatic structures is responsible for the peak at 2905 cm^−1^ [29]. On the other hand, the peak at 1492 cm^−1^ is caused by the C-H bond bending, reflecting the presence of alkanes [30]. Both of these peaks are contributed by rec-HDPE. However, at a wavenumber of 955 cm^−1^, peaks are observed only in the rice husk-derived composites and are absent in the other two material’s FTIR spectra. These peaks are actually caused by the C-O stretching vibration in the rice husk-reinforced composites, indirectly indicating the absence of C-O stretching vibration in rec-HDPE as well as wood flour [31]. This C-O stretching vibration occurs due to the presence of non-starch and starch carbohydrates in rice husk. Similarly, the minor peak at 3623 cm^−1^ is caused by the O-H bond stretching vibration and is also contributed by rice husk only [32,33]. The peaks that are contributed only by the wood flour are at wavenumbers of 3417 cm^−1^ and 1767 cm^−1^. The former peak is caused by the O-H stretching, and the latter one is caused by the C=O stretching of the Carbonyl and Ester groups [34]. This study also demonstrates that these functional groups were influenced by rec-HDPE, wood flour, or rice husk, indicating physical and chemical interaction between them.

### 3.2. Tensile Properties

**Figure 3:**
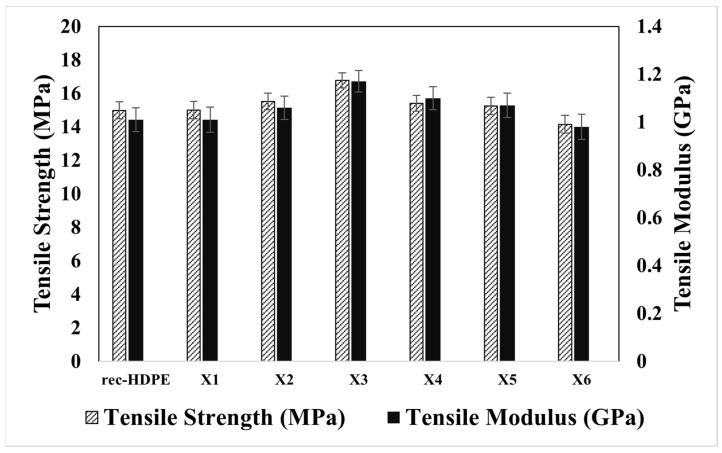
Tensile properties of all materials.

Figure 3 represents the tensile properties of the composites and the rec-HDPE. The tensile strength of rec-HDPE was evaluated to be ~14.99 MPa, and the tensile modulus was found to be ~1.01 GPa. The tensile strength of the composite samples (rec-HDPE, X1, X2, X3, X4, X5, and X6) was found to be 14.99, 15.01, 15.52, 16.77, 15.41, 15.25, and 14.15 MPa, respectively, while the tensile modulus was found to be 1.01, 1.01, 1.06, 1.17, 1.1, 1.07, and 0.98 GPa, respectively. Composite X3 (10% rice husk-filled rec-HDPE) has displayed the highest tensile properties among all the composites. The tensile strength and tensile modulus of the X3 composite are 11.9% and 15.8% higher than that of rec-HDPE, respectively.

The mechanical properties of rice husk and wood flour-reinforced composites depend on several factors. The concentration of rice husk reportedly affects the mechanical properties of composites [35,36]. Increased rice husk concentration tends to enhance the stiffness of rec-HDPE [37]. The enhanced interfacial bonding of the fillers and rec-HDPE leads to good stress propagation, resulting in enhanced tensile strength. Similar results regarding interfacial bonding have been reported in the literature [38]. In this study, MAPE played a role as a compatibilizer to enhance the mechanical properties of the composites. A compatibilizer can enhance the interfacial bonding of the filler and matrix [21,39]. The mechanical properties of composites are also controlled by the uniform distribution of filler nanoparticles in the polymer and the degree of agglomeration of particles with each other [40]. If particles are uniformly dispersed in the polymer matrix, it would result in greater tensile strength, while agglomeration and clustering of filler particles lead to a decrease in tensile strength. Composites with rice husk as a single filler have displayed higher tensile properties as compared to those with hybrid fillers (rice husk and wood powder). This is mainly because of the combined effect of the two fillers.

### 3.3. Scanning Electron Microscopy (SEM)

**Figure 4:**
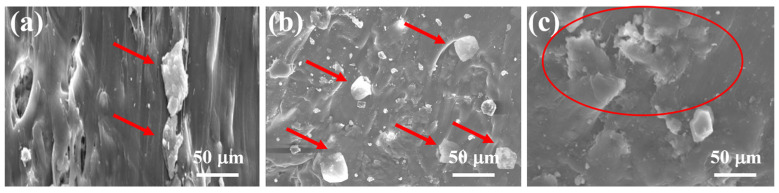
SEM images of (**a**) X1, (**b**) X3, and (**c**) X6 composites.

The SEM micrographs of the fractured surfaces of X1, X3, and X6 after the tensile test are shown in Figure 4. The images highlight minor agglomeration of particles (as shown by arrows in Figure 4a) in the X1 composite, which contains 5% wood flour and 5% rice husk in rec-HDPE. The X3 sample, comprised of 10% rice husk and rec-HDPE, exhibited no agglomeration and better particle distribution (as shown by arrows in Figure 4b). This composite displayed the highest tensile properties. No debonding between rice husk and rec-HDPE can be seen in the SEM image of X3, indicating better interfacial bonding in the presence of MAPE. The SEM image of X6 also shows agglomeration and poor distribution of the particles in the matrix (as shown by the circle in Figure 4c); this specimen contains 20% wood flour in rec-HDPE. This led to decreased tensile strength in X6 compared to X3. Overall, it has been observed that wood flour particles tend to agglomerate, whereas rice husk particles exhibit better dispersion. This is why rice husk-filled rec-HDPE composites have shown higher tensile properties compared to wood flour and hybrid particles-filled rec-HDPE composites.

### 3.4. Flexural Properties

**Figure 5:**
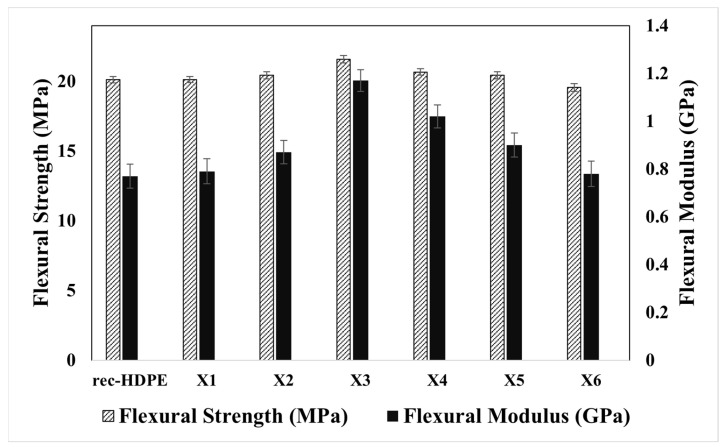
Flexural properties of all materials.

Figure 5 shows the flexural properties of the materials developed in this study. The flexural strength of rec-HDPE was found to be ~20.12 MPa. The flexural strength of rec-HDPE, X1, X2, X3, X4, X5, and X6 was found to be 20.12, 20.14, 20.44, 21.59, 20.67, 20.44, and 19.56 MPa, respectively. Among all the samples, X3 exhibited the maximum flexural strength, showing an enhancement of about 7.3% compared to that of pure recycled HDPE. The flexural modulus of rec-HDPE was discovered to be 0.77 GPa. The flexural modulus of the composites (X1, X2, X3, X4, X5, and X6) was found to be 0.79, 0.87, 1.17, 1.02, 0.9, and 0.78 GPa, respectively.

Flexural properties of polymer composites may exhibit enhancement with the inclusion of various filler content up to a certain limit [41,42]. This is due to the increase in the volume fraction of high modulus lignocellulosic content in the composite [38]. Compatibilizer also affects flexural strength. In this study, the compatibilizer (MAPE) may be responsible for enhancing the interfacial bonding between rec-HDPE and the filler particles. The size of filler particles also influences flexural properties. Wood flour particles were larger in size than rice husk, so wood flour-reinforced rec-HDPE composites possessed less flexural strength than rice husk-reinforced rec-HDPE composites. The better distribution of rice husk particles in rec-HDPE and agglomeration in the case of wood flour and hybrid particles-filled rec-HDPE composites has already been reported in the SEM analysis, which is also reflected in the flexural test results.

### 3.5. Charpy Impact Test

**Figure 6:**
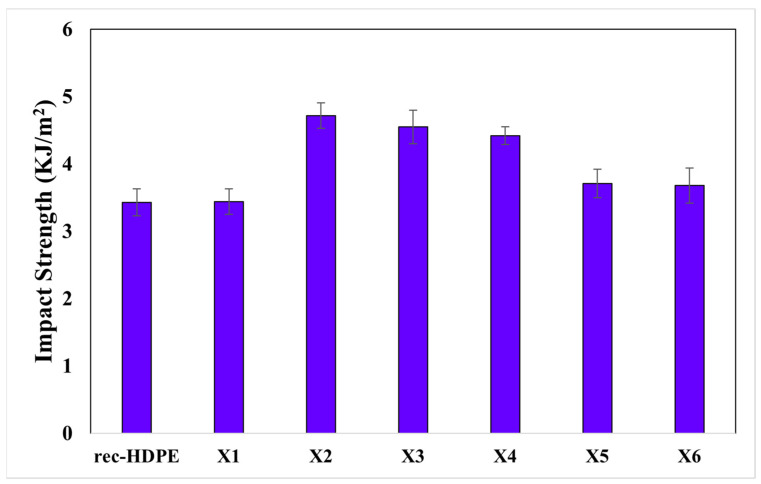
Impact properties of all materials.

The impact strength of all samples is shown in Figure 6. The impact strength of recycled HDPE was discovered to be 3.43 KJ/m^2^. The impact strength of the composite samples (rec-HDPE, X1, X2, X3, X4, X5, and X6) was found to be 3.43, 3.44, 4.72, 4.55, 4.42, 3.71, and 3.68 KJ/m^2^, respectively. The impact strength displayed a different trend compared to tensile strength and flexural strength. Impact strength is defined as the capability of the material to absorb energy due to a sudden load applied to it before fracture. The impact strength of the X2 composite was found to be the maximum in this study. This composite contains hybrid fillers (10% wood flour and 10% rice husk) reinforced in rec-HDPE. The effect of the hybrid particles has caused an increase in the impact strength due to a higher coefficient of friction between the unidentical particles, leading to increased resistance against impact loading. Similar results regarding the synergetic effects of hybrid fillers in polymer composites have been reported in the literature [43,44]. Table 2 compares the mechanical properties of all materials.

### 3.6. Horizontal and Vertical Burning Tests

All material specimens underwent horizontal and vertical burning tests. Five specimens were tested for each material type in both tests, and the average of the results was calculated. Table 3 shows the results of the burning tests. In the case of horizontal burning tests, the burning rates of all the specimens were higher than 40 mm/min, except X5, which received an HB rating owing to its best burning rate of 37.25 mm/min. All specimens containing filler particles displayed lower burning rates compared to rec-HDPE. Moreover, the burning rates of rice husk-filled rec-HDPE composites were lower than those of wood flour-filled rec-HDPE. The decrease in the average burning rate from rec-HDPE (45.10 mm/min) to X5 (37.25 mm/min) was recorded as 17.40%.

Regarding the vertical burning tests, no material except the X5 composite received any rating, particularly because of the cotton ignition by the flaming drops and the flame that reached the holding clamp. Rec-HDPE did not receive any rating due to its poor flame retardancy characteristics, which have also been reported in the literature [45]. Only the X5 composite was able to obtain a V-2 rating, owing to the higher loading of rice husk content, which prevented the flame from reaching the holding clamp. Moreover, the after-flame time of the composites that involved wood flour was comparatively longer than that of rec-HDPE.

### 3.7. Cone Calorimeter Test

**Figure 7:**
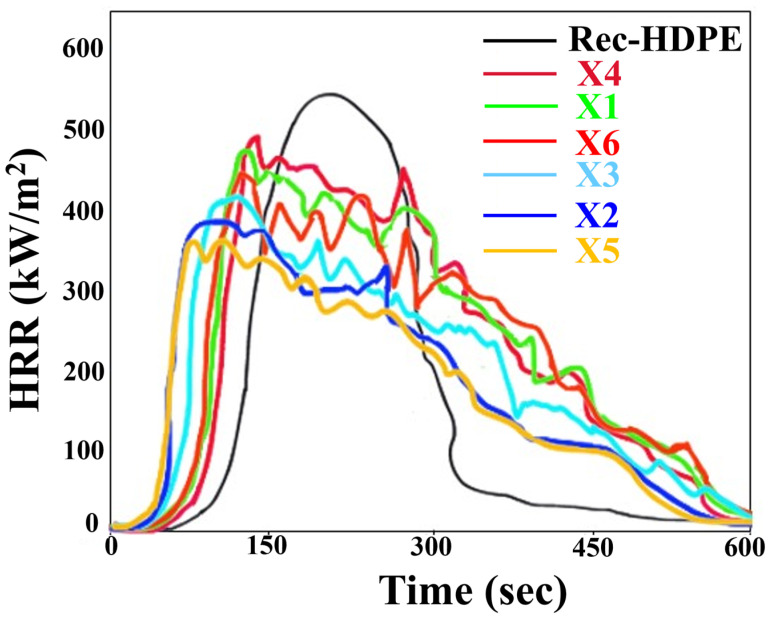
Heat release rate of all types of samples.

**Figure 8:**
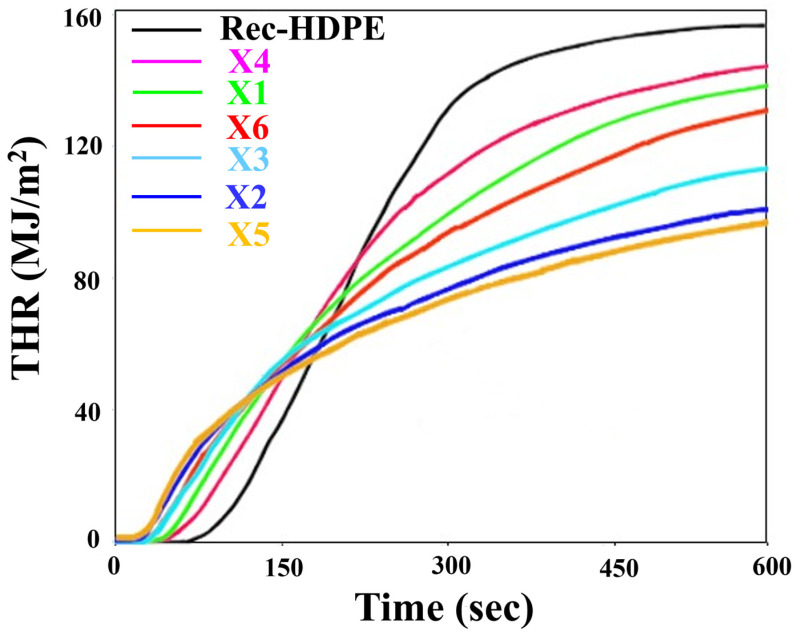
Total heat release rate of all types of samples.

Cone calorimetry was carried out to better understand and explain the fire-retardant properties of recycled HDPE as well as other composite samples. Table 3 shows the values for the total heat release rate (THR) and peak heat release rate (PHRR) of all material samples, while the heat release rate (HRR) and THR have been plotted against time in Figure 7 and Figure 8, respectively.

The PHRR and THR of recycled HDPE were 548.66 kW/m^2^ and 155.4 MJ/m^2^, respectively, due to its poor flammability and vigorous burning. Both parameters decreased when the combined effect or individual effects of rice husk and wood flour loading on rec-HDPE were observed. X5 composite showed the lowest PHRR and THR values, indicating its enhanced fire retardancy. Compared to those of rec-HDPE, X5’s PHRR and THR values both dropped by 33.78% and 38.06%, respectively. Rice husk-filled rec-HDPE exhibited better flame-retardant characteristics than wood flour-filled rec-HDPE composites when compared at the same filler loadings. Moreover, the hybrid fillers-based composites also showed improved fire-retardant properties compared to wood flour-based composites, demonstrating the combined effect of hybrid fillers on the fire-retardant properties of these materials. Based on the cone calorimetry, the trend for the best fire-retardant characteristics is as follows: X5 > X2 > X3 > X6 > X1 > X4 > rec-HDPE.

Liu et al. comprehensively addressed the development, application, and problems of conventional flame-retardant methods [46]. In the current investigation, the reason behind the maximum increase in fire-retardant properties of the rice husk-based composite (X5) is that it contains a high amount of inorganic silica [12]. During combustion, all the organic components are gasified, whereas silica remains the only residue left behind. Its continuous accumulation results in the formation of a silica ash layer, which plays a prominent role as a barrier against heat [12,47,48].

### 3.8. Limited Oxygen Index Test (LOI Test)

The LOI experiments were used to elucidate the flame-retardant behavior of rec- HDPE as well as rice husk and wood flour-reinforced composite materials. LOI is the volume ratio of oxygen in a mixture of nitrogen and oxygen. It represents the smallest amount of oxygen required to sustain a flame. The greater the limited oxygen index of a specimen, the better its flame-retardant properties.

**Figure 9:**
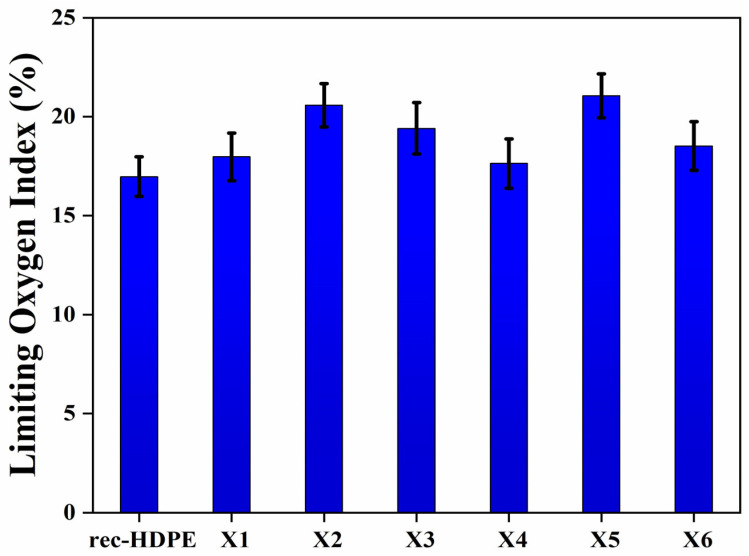
Limited oxygen index of all types of samples.

Results of the LOI test are shown in Figure 9, and it can be seen that the LOI of rec-HDPE was the lowest among all samples, but its value increased for all other samples. The rice husk-based composites exhibited higher values of LOI than the wood flour-based composites when compared at the same filler loadings. Rec-HDPE showed an LOI value of around 16.97%, whereas the LOI of X5 came out to be 21.06%, which is the highest among all. The prime reason behind the enhancement in LOI of the composites is the presence of wood flour and rice husk in these materials and its mechanism is the same as discussed in the previous section. Due to the decomposition of cellulose, hemicellulose, and lignin in these biomasses, silica oxides form a protective layer around the composites, reducing heat transfer and improving LOI. This leads to enhancing the flame-retardant characteristics of these composites [12,47,48]. Table 3 reveals the results of the LOI for all samples.

### 3.9. Thermogravimetric Analysis (TGA)

**Figure 10:**
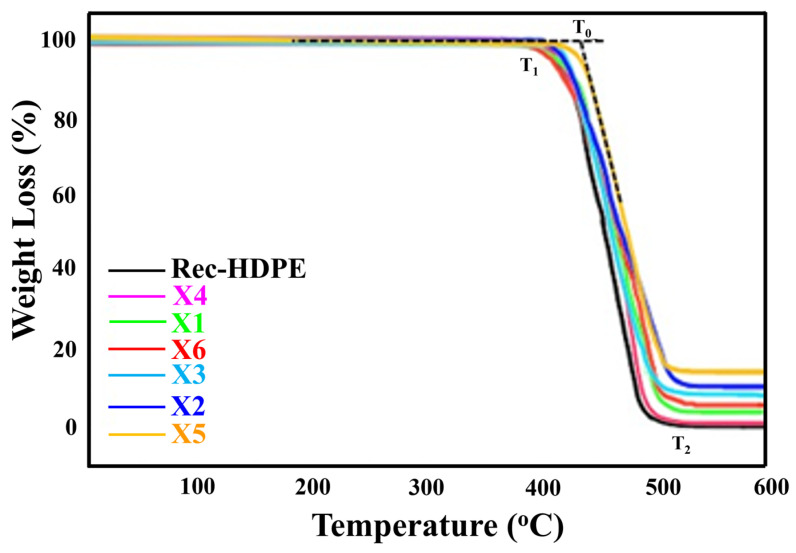
TGA curves of all types of samples.

Figure 10 depicts the TGA curves of all the materials between room temperature and 600 °C. These curves reveal that as the temperature was raised, these samples initially responded effectively with no degradation. However, as the temperature hit 402.34 °C, the rec-HDPE sample began to thermally degrade, with degradation continuing until 522.16 °C. The X5 composite was the last one to decay with an onset degradation temperature of 436.13 °C and a final degradation temperature of 536.74 °C. An increase in the onset degradation temperature (T_0_), starting degradation temperature (T_1_), and final degradation temperature (T_2_) was recorded for all composites compared to those of rec-HDPE. The rice husk-reinforced composites had greater thermal stability than the wood flour-reinforced composites when compared to the same filler content [49]. With reference to the above discussion, rice husk was a better filler in terms of improving thermal stability than wood flour.

The char residue left at 600 °C was also the maximum for the X5 composite, which revealed the improvement in thermal stability. The reason behind the enhancement of thermal stability was the same as the increase in flame-retardant properties. Silica oxides in rice husk-reinforced composites formed a protective layer around the composite due to the decomposition of its constituents in response to combustion. This protective layer acted against thermal degradation and thus improved the overall thermal stability [12,47,48]. Hybrid fillers-based composites showed enhanced thermal stability compared to wood flour-filled composites at the same filler loading. Table 4 shows T_0_, T_1_, T_2_, and residue % of char at 600 °C for all materials studied. The order of thermal stability from highest to lowest was found as X5 > X2 > X3 > X6 > X1 > X4 > Recycled HDPE.

## 4. Conclusions

In this study, the synergistic and individual effects of rice husk and wood flour on the properties of rec-HDPE were investigated. Mechanical, thermal, and fire-retardant properties were studied with different combinations of filler content.

Maximum tensile and flexural properties were found for rec-HDPE filled with 20% rice husk. However, impact strength was found to be maximum with hybrid reinforcement of rice husk and wood flour (10% each) in the rec-HDPE. The increase in mechanical properties was due to the uniform distribution of filler particles and less agglomeration of filler particles in the polymer matrix, which was revealed by SEM analysis as well. The coupling agent in composites also played a vital role in enhancing mechanical properties as it improved the interfacial bonding between the filler particles and the polymer matrix.

Thermal stability and flame retardancy of composites also increased after the addition of filler particles in rec-HDPE. These properties were studied using thermogravimetric analysis, burning tests, cone calorimetry, and limited oxygen index tests. Rice husk (20%) was found to have a greater effect on fire retardancy and thermal stability than wood flour. The formation of a silica layer due to the decomposition of cellulose, hemicellulose, and lignin in these fillers resulted in greater thermal stability and flame retardancy, preventing further decomposition of the composite material. Further evaluation of the insulation and dielectric properties of the material developed in this study may be conducted for specific applications. Rec-HDPE could be used in cable trays and electric boards, and these composites of rec-HDPE are expected to have enhanced insulation properties.

## Figures and Tables

**Table 1:** Nomenclature of the composite samples.

Sr. No	Recycled HDPE Pellets wt. %	Rice Husk Powder wt. %	Wood Flour Powder wt. %	Compatibilizer (MAPE) wt. %	Nomenclature
1	100	0	0	0	rec-HDPE
2	87	5	5	3	X1
3	77	10	10	3	X2
4	87	10	0	3	X3
5	87	0	10	3	X4
6	77	20	0	3	X5
7	77	0	20	3	X6

**Table 2:** Mechanical properties of all material samples.

Samples	Tensile Strength (MPa)	Tensile Modulus (GPa)	Flexural Strength (MPa)	Flexural Modulus (GPa)	Impact Strength (kJ/m^2^)
rec-HDPE	14.99	1.01	20.12	0.77	3.43
X1	15.01	1.01	20.14	0.79	3.44
X2	15.52	1.06	20.44	0.87	4.72
X3	16.77	1.17	21.59	1.17	4.55
X4	15.41	1.1	20.67	0.02	4.42
X5	15.25	1.07	20.44	0.9	3.71
X6	14.15	0.98	19.56	0.78	3.68

**Table 3:** Fire retardant properties of all types of samples.

Cone Calorimetric Test					Horizontal Burning Test				Vertical Burning Test					LOI Test
Types	TTI (sec)	TPHRR (sec)	PHRR (kW/m^2^)	THR (MJ/m^2^)	Avg. Burning Time (Min)	Avg. Burning Rate (mm/Min)	Rating	Max. after Flame Time (sec)	Total after Flame Time (sec)	Max after Flame + after Glow Time (sec)	Flame up to the Holding Clamp	Cotton Ignited by Flaming Drops	Rating	LOI (%)
**Rec-HDPE**	59	210	548.66	155.40	1.66	45.10	Nil	61	281	-	Yes	Yes	Nil	16.97
**X1**	46	130	475.76	137.31	1.77	42.22	Nil	53	261	-	Yes	Yes	Nil	17.97
**X2**	36	96	389.25	100.22	1.83	40.84	Nil	50	252	-	Yes	Yes	Nil	20.58
**X3**	36	123	418.91	112.49	1.82	41.06	Nil	52	257	-	Yes	Yes	Nil	19.41
**X4**	51	138	494.29	143.14	1.70	44.04	Nil	55	271	-	Yes	Yes	Nil	17.63
**X5**	33	104	363.3	96.24	2.01	37.25	HB	24	237	56	Yes	No	V-2	21.06
**X6**	45	127	446.10	129.65	1.86	40.18	Nil	48	235	-	Yes	Yes	Nil	18.52

**Table 4:** Thermal properties of the recycled HDPE and composites.

Sample	T_o_ (Onset Degradation Temperature) (°C)	T_1_ (Starting Degradation Temperature) (°C)	T_2_ (Final Degradation Temperature) (°C)	Residue % at 600 °C
REC-HDPE	426.55	402.34	522.12	0.39%
X1	431.38	404.97	525.95	3.93%
X2	429.94	409.29	533.08	10.66%
X3	427.84	407.64	531.32	8.91%
X4	426.74	402.92	525.88	0.95%
X5	436.13	413.11	535.19	14.73%
X6	432.86	403.55	536.74	6.81%

## Data Availability

All data are included in the manuscript.
